# Establishing an astaxanthin-rich live feed strain of *Pseudodiaptomus annandalei*

**DOI:** 10.1038/s41598-024-59224-y

**Published:** 2024-04-15

**Authors:** Sen Chan, Yen-Ju Pan, Ang Lu, Chang-Wen Huang, Ji-Long Liao, Jui-Sheng Chang, Gaël Dur

**Affiliations:** 1 Department of Aquaculture, National Taiwan Ocean University, Keelung, Taiwan, R.O.C.; 2 Center of Excellence for the Oceans, National Taiwan Ocean University, Keelung, Taiwan, R.O.C.; 3 Creative Science Unit (Geoscience), Faculty of Science, Shizuoka University, Shizuoka, Japan

**Keywords:** Marine biology, Animal physiology, Liquid chromatography

## Abstract

This study aimed to establish an astaxanthin-rich strain of the calanoid copepod *Pseudodiaptomus annandalei*, through selective breeding based on RGB (red, green and blue) value, a parameter indicating color intensity. We evaluated the RGB value frequency distributions of the copepod populations, and selected individuals with the highest 10% and the lowest 10% RGB value over six generations. The RGB value, nauplii production, clutch interval and clutch number were assessed, and the genetic gain was calculated across generations (G_0_-G_5_). Two strains of copepods were selected and defined as dark body copepod strain (DBS) and light body copepod strain (LBS) at the end of experiment. Results revealed significantly lower RGB values (male: 121.5 ± 14.1; female: 108.8 ± 15) in the G_5_ DBS population compared to the G_0_ (male: 163.9 ± 13.1; female: 162.2 ± 14.6), with higher genetic gains of RGB values during G_0_ to G_2_. While DBS females exhibited longer clutch intervals in the G_3_ and G_4_, there was no significant difference in nauplii production between the two strains across all generations. Significantly higher astaxanthin content was found in the DBS copepods (0.04 μg/ ind.) compared to the LBS copepods (0.01 μg/ ind.) and the non-selective copepods (0.02 μg/ ind.) 20 months post selective breeding, validating the stability of the desired trait in the DBS strain. This study successfully established an astaxanthin-rich strain of *P. annandalei*, which provides implications for enhancing marine and brackish larviculture production.

## Introduction

Copepods, a group of small zooplankton inhabiting diverse aquatic environments, play a pivotal role in coastal ecosystems, often constituting over 75% of the total zooplankton community^1^. As an essential component of marine mesozooplankton, copepods substantially influence marine trophic systems. Studies investigating gut contents of several fish larvae, including silver pomfret (*Pampus argenteus*), bluefin tuna (*Thunnus thynnus*), marbled rockfish (*Sebastiscus marmoratus*), and sand lance (*Ammodytes japonicus*), underscored the significant role of copepods as natural prey items for fish larvae^2–4^. Copepods have been employed as live feed for aquatic larvae since the 1980s^5^, leading to enhanced aquaculture performance in larval rearing, and supporting the cultivation of many emerging marine fish species^6–8^.

Copepods offer distinct advantages over traditional live feeds like *Artemia* and rotifers. Notably, their captivating “jerking” swimming pattern fosters higher larval predation^9,10^. Their small body size (< 100 μm) renders them suitable for feeding small-mouthed fish larvae^11,12^. Most significantly, copepods are nutritionally rich in long-chain polyunsaturated fatty acids (LC-PUFAs), amino acids, pigments, and various micronutrients^13,14^. LC-PUFAs, particularly eicosapentaenoic acid (EPA) and docosahexaenoic acid^15^, are pivotal for the survival, growth, development, and stress resilience of many marine larvae^16,17^. Certain copepod species demonstrate the capacity to convert short-chain fatty acid into LC-PUFA through biochemical precursors from dietary microalgae. The species *Pseudodiaptomus annandalei*, a prevalent calanoid copepod in the aquaculture industry, was noted for its LC-PUFA bioconversion ability even under PUFA-deficient dietary conditions^18,19^. Other copepod species, such as *Apocyclops royi*^20^ and *Tigriopus californicus*^21^, also exhibit similar LC-PUFA bioconversion capacity.

Astaxanthin, the most dominant form of carotenoid pigments in copepods^14,22^, is correlated with their body color^23,24^. RGB value has been utilized as an indicator for carotenoid content in aquatic animals^25,26^. Although the function and causation of astaxanthin accumulation in copepods are not fully understood, studies suggested its role in protecting against oxidative stresses induced by ultraviolet radiation^27^ and metal toxicants^28^. Additionally, high astaxanthin content in copepods might serve to safeguard their abundant LC-PUFAs content in lipid droplets^29^. As a potent antioxidant, astaxanthin has several nutritional benefits to aquatic animals^30^. Dietary supplement of astaxanthin could improve the immunity, reproduction and pigmentation of juvenile and adult fish^31–33^. For larviculture, supplement of dietary astaxanthin could also support higher larval survival, growth rate, and stress resistance^34,35^. However, the deficiency in astaxanthin in rotifers and *Artemia,* necessitates additional enrichment protocols for feeding fish larvae^36^. Such enrichment, often relying on microalgae based or synthetic astaxanthin, can be cumbersome and costly, with the bioavailability of astaxanthin diminishing over time during storage and digestion within the rotifer and *Artermia*. In contrast, esterified astaxanthin accumulated in copepods remains relatively stable and bioavailable^37,38^. Copepod species or strains with high astaxanthin contents, especially when coupled with their high LC-PUFAs content, may offer the potential to significantly enhance marine larviculture production.

This study concentrates on the calanoid copepod *P. annandalei*, prevalent in tropical and subtropical coastal ecosystems across the Indo-Pacific region^39–41^. This euryhaline egg-bearing species of *Pseudodiaptomus*, as well as its congener species, is commonly cultivated on a commercial scale^15,42–44^, and harvested for larval feeding in aquaculture industry^45,46^. However, the inter-individual variability of color-associated traits has rarely been investigated. Moreover, no study has specifically focused on selective breeding for traits associated with astaxanthin content in copepods. In this context, we aimed to establish an astaxanthin-rich strain of *P. annandalei* through selective breeding, evaluating the interrelations among body color, astaxanthin content, and copepod productivity.

## Materials and methods

### Microalgae and copepod stock culture

The pure strain of microalga and copepod employed in this study were obtained from the Tungkang Biotechnology Research Center, Fisheries Institute of Taiwan. The stock cultures of microalgae and copepod were maintained following the protocol described by Beyrend-Dur et al^39^. The microalga *Isochrysis galbana* was cultivated in 16 L polycarbonate carboys, utilizing 1-μm filtered, sterilized, and aerated seawater maintained at a salinity of 35 ppt. The Walne medium^47^served as the nutritional source, with cultures re-inoculated every 10–14 days. The stock culture of the copepod *P. annandalei* was maintained in 20 L polycarbonate carboys, using 1-μm filtered and aerated seawater at a salinity of 20 ppt, achieved through a mixture of natural seawater and distilled water. The copepods were fed with microalga *I. galbana* every two days at an approximate cell concentration of 10^5^ cells/ mL. A complete water exchange of the stock culture was conducted every two weeks to maintain the water quality. The microalga and copepod cultures were maintained at a constant temperature of 28 ± 1 °C. The photoperiod employed was a balanced 12-h light: 12-h dark cycle, with light intensity set at 4000 lx for microalgae and at 400 lx for copepod.

### Identification of astaxanthin in *P. annandalei*

To establish the protocols for the identification and quantification of astaxanthin content in *P. annandalei*, a comprehensive series of tests was conducted. To mitigate potential overestimation due to gut residues, all copepods were subjected to gut evacuation prior to extractions. Lyophilized copepods (approximately 1 mg) were placed in a 15 mL centrifugation tube containing 90% acetone, followed by a 2-min sonication and overnight storage at 4 °C in the dark. After extraction, 10 mM NaOH was introduced into the tube for a 2-h saponification process at room temperature. The resultant extractant underwent filtration via a 0.22 μm syringe filter before further analysis. To evaluate the astaxanthin content per individual copepod, 10 adult copepods were carefully sorted and placed in the microcentrifuge tube with 200 uL 90% acetone, and subjected to the extraction and saponification as aforementioned. The astaxanthin extractant was collected from the supernatant after 10-min centrifugation at 8,000 rpm.

The optimal wavelength for detecting copepod astaxanthin and the free astaxanthin standard was examined by conducting a spectrum scan ranging from 380 to 520 nm using the spectrophotometer CT-2200 (ChromTech, Singapore). Subsequently, serial dilutions of astaxanthin standard solutions (0.1, 0.25, 0.5, 0.75, 1, 1.5, 2 μg/mL, 3S 3′S-astaxanthin standard, Sigma-Aldrich, USA) were prepared and measured at the optimal wavelength to establish a correlation between absorbance and astaxanthin concentration.

The extract was injected to High Performance Liquid Chromatography (HPLC) system (Jasco Inc, Tokyo, Japan) equipped with Agilent XDB-C18 column (5 μm, 250 × 4.6 mm, Agilent Technologies, California, USA). A flow rate of 1 mL min^−1^ was employed, following a linear gradient from 100% A (80:20, methanol: 0.5 M ammonium acetate) to 100% B (90:10, acetonitrile: H_2_O) over 4 min, followed by a transition to 20% B: 80% C (ethyl acetate) over 14 min. The gradient then returned to 100% B over 3 min before ultimately returning to 100% A over 5 min, and held for 6 minutes^48^. Signal of the absorbance was measured at 482 nm using an ultraviolet UV–visible detector. The chromatographic characteristic of the copepod extract was compared to the (3S, 3’S)-astaxanthin standard.

### Imagery analysis of copepod body color

For the purpose of selective breeding on color-associated traits, the value of RGB (red, green and blue) composition was evaluated through a meticulous analysis of copepod digital images. To prevent any potential bias from gut content coloration, all copepods underwent a 24-h gut evacuation treatment prior to photographing. Subsequently, copepods were placed individually on cavity slides and photographed using a Motic BA210 compound microscope (Motic, China), maintaining consistent optical settings (magnification 100X, fixed condenser and light intensity). After photography, the copepod individuals were immediately transferred to a 12-well culture plate (3.5 mL/ well), where it was temperately cultivated for further selection. Simultaneously, the RGB value corresponding to the prosome and urosome areas, where the pigments primarily accumulated, was analyzed using Adobe Photoshop CS6 software (Adobe Systems, CA, USA) following the protocol described by Díaz‐Jiménez et al^25^. This approach enabled a rapid assessment of the RGB values for living copepod individuals with no mortality caused, serving as an indicator of the astaxanthin-associated trait for selective breeding in the present study.

### Selective breeding protocol based on copepod body color

**Figure 1:**
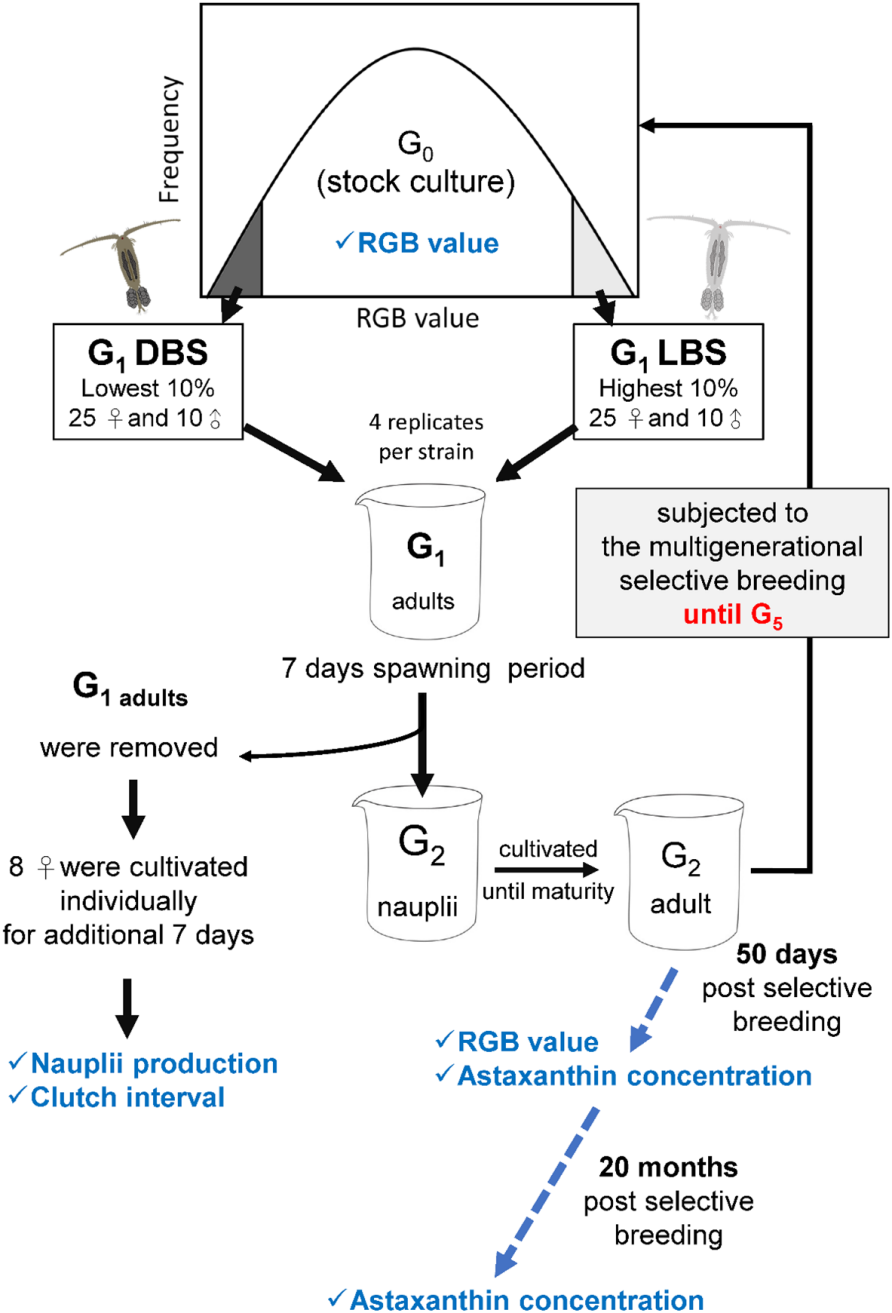
Protocol of selective breeding for *P. annandalei* light, and dark body strains (LBS, DBS) based on RGB value.

The stepwise procedure for the selective breeding protocol is visually presented in Fig. 1. To initiate the process, a representative cohort of 40–50 adult copepods was randomly collected from the stock culture (G_0_), and their body colors were subjected to imagery analysis as described in section "Imagery analysis of copepod body color". Frequency distributions of RGB value were constructed immediately after imagery analyses for males and females, respectively. Based on the RGB frequency distributions of the G_0_ population, 25 females and 10 males with the highest 10% RGB values (i.e. the 10% lightest) and the lowest 10% RGB values (i.e. the 10% darkest) were then selected, and transferred to 2L beakers to initiate the cultures of the first selective generation (G_1_) for light body strain (LBS) and dark body strain (DBS), respectively.

The selected G_1_ copepods were cultivated in 2L beakers (4 replicates) containing diluted seawater at 20 ppt and maintained at 28 ± 1 °C. After a 7-day spawning duration (approximately 3 clutches), the G_1_ adults were removed from the beakers through sieving using a 500 μm mesh. The nauplii and copepodites of the subsequent generation (G_2_) were cultivated until maturity. The cultures were observed daily, and the subsequent selection was conducted 24 h after the first appearance of ovigerous females in the populations. A group of 40–50 G_2_ adult copepod was collected from every population, and subjected to the imagery analysis, and the selection of G_2_ population was conducted as aforementioned. The multigenerational selective breeding protocol was carried out repetitively for 5 successive generations (G_1_ to G_5_).

To assess the variation of female productivities between the LBS and DBS strains across generations, eight ovigerous females per replicate were collected at random from the removed adults after the 7-day spawning duration. These females were independently cultivated in 12-well culture plates containing 3.5 ml of culture water. The nauplii production and clutch interval were documented daily under a stereomicroscope (SZX9, OLYMPUS, Tokyo, Japan) over a 7-day span. In this study, all copepod individuals were gently transferred using wide-bore pipettes, and no mortality was observed during the manipulations.

Genetic gain, a measure of proportional phenotypic alteration during selective breeding, was calculated as the percentage change in the evaluated biological trait between successive generations. The genetic gain in RGB value was quantified using the formula:

$${\text{Genetic gain}} = \left( {{\text{RGB}}_{{({\text{Gx}})}} - {\text{RGB}}_{{({\text{Gx}} - {1})}} } \right)/\left( {{\text{RGB}}_{{({\text{Gx}} - {1})}} } \right)*{1}00\;\%$$

where RGB_(Gx)_ represents the RGB value of the present generation; RGB_(Gx−1)_ represents the RGB value of the preceding generation.

The actual astaxanthin concentrations (per copepod biomass and individual) and RGB values of both LBS and DBS copepods were ascertained 50 days post selective breeding (PSB). In addition, the actual astaxanthin concentrations of the LBS, DBS and stock culture (the non-selective population) were analyzed 20 months post the selective breeding program. These assessments served to determine the stability of the desired trait within the selective copepod strains.

### Data analysis

To evaluate the differences between the two selective strains at the same generation, comparisons of RGB values, astaxanthin content, and productivity were performed using the student’s t-test. Furthermore, for analyzing data among the five generations and three populations (LBS, DBS and stock culture), a one-way ANOVA was employed. Subsequent to the identification of significant differences, the Tukey multiple comparison test was conducted to elucidate their pairwise differences between groups. All statistical analyses were executed utilizing SPSS 18.0 (SPSS, Chicago, IL, USA), with the predetermined significant level set at *p* < 0.05.

## Results

### Identification and quantification of astaxanthin in *P. annandalei*

**Figure 2:**
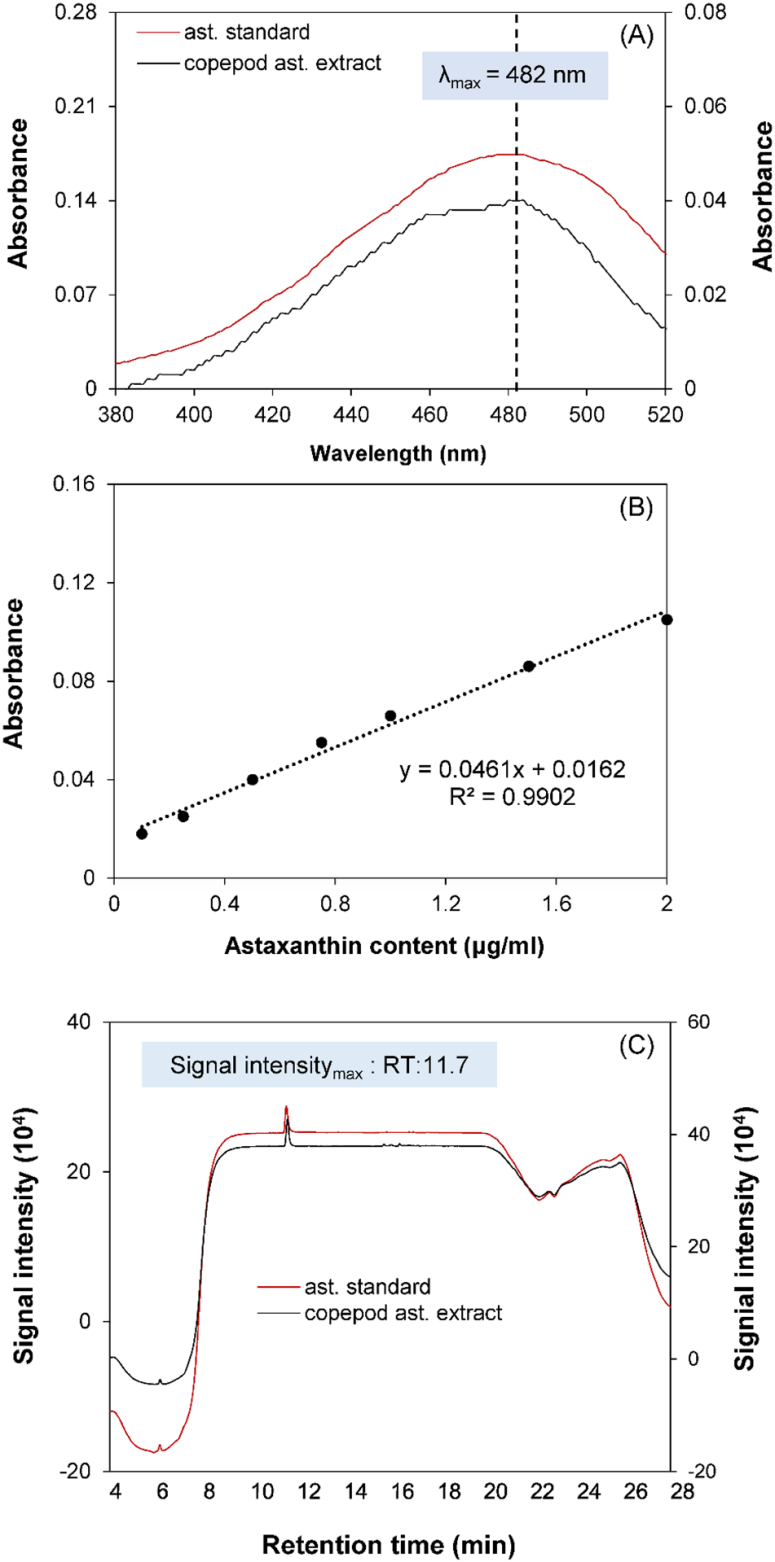
(**A**) Absorbance of the astaxanthin standard (left axis) and *P. annandalei* astaxanthin extract (right axis) in the wavelength spectrum of 380–520 nm; (**B**) Correlation between absorbance at 482 nm and concentration of astaxanthin standard; (**C**) HPLC chromatograms of astaxanthin standard (left axis) and *P. annandalei* astaxanthin extract (right axis). ast.: astaxanthin.

The maximum absorbance for both copepod astaxanthin and the astaxanthin standard was recorded at 482 nm (Fig. 2A). A clear and consistent linear relationship was observed between astaxanthin concentration and absorbance (Fig. 2B). The equation coefficient of determination value (R^2^) was calculated to be 0.9902.

The HPLC chromatogram of the copepod pigment extract displayed a prominent peak at a retention time of 11.7 min (Fig. 2C). The chromatographic characteristic and retention time of the extracted astaxanthin were identical to those of the free (3S, 3′S)-astaxanthin standard (Fig. 2C).

### Selective breeding based on copepod body color

#### RGB value of copepod body

**Figure 3:**
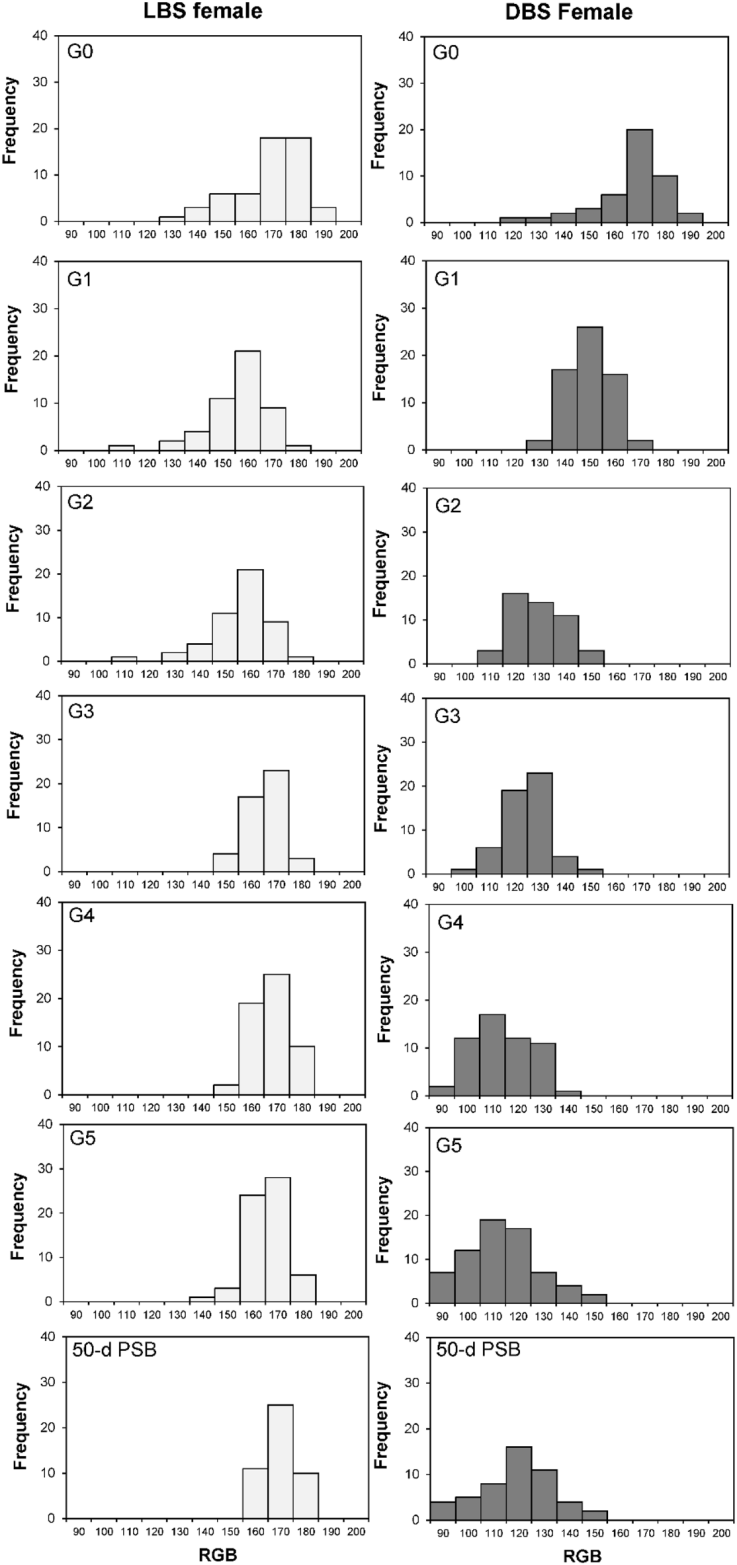
Frequency distribution of RGB value of the *P. annandalei* females in the LBS and DBS populations across generations. PBS: post selective breeding.

**Figure 4:**
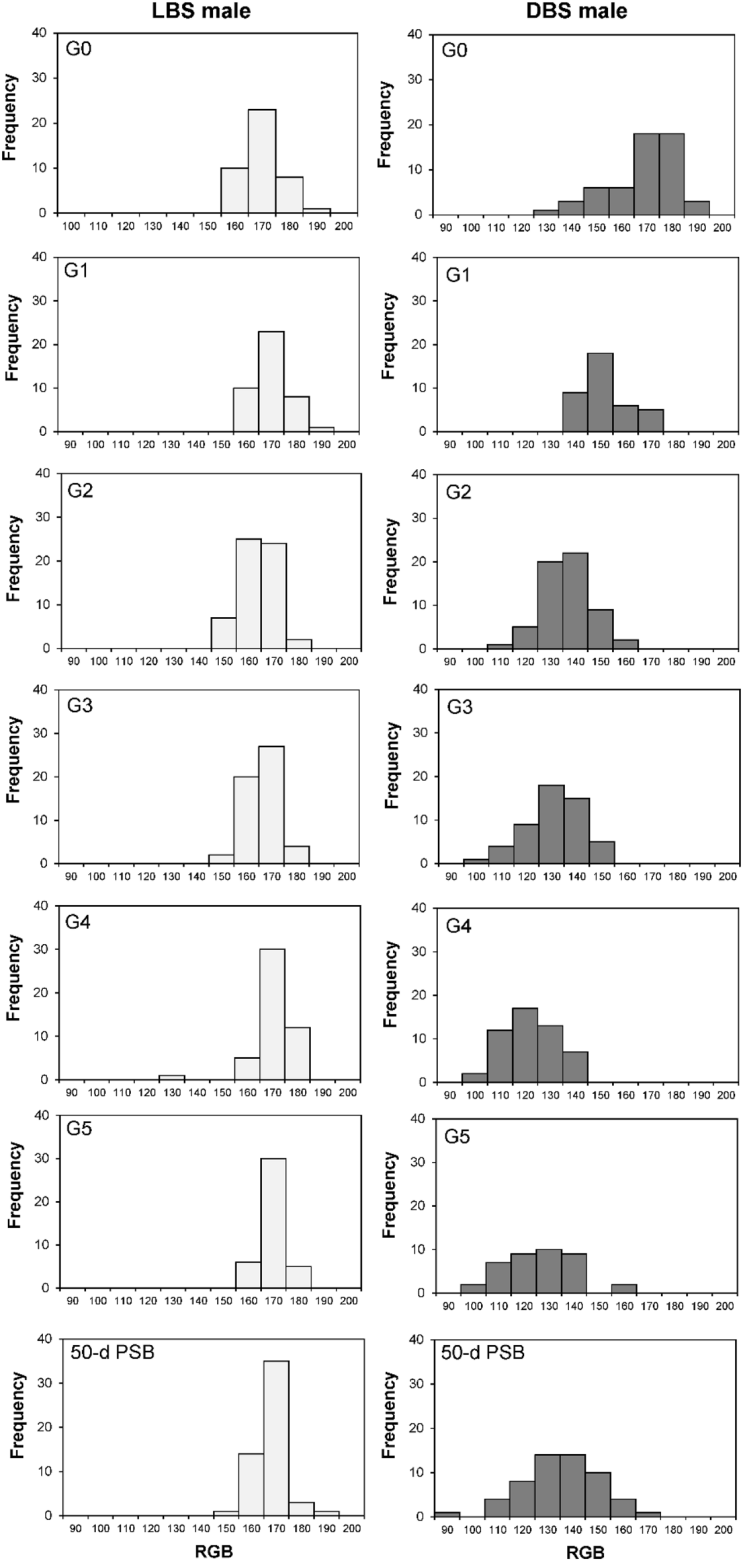
Frequency distribution of RGB value of the *P. annandalei* males in the LBS and DBS populations across generations. PBS: post selective breeding.

Within the LBS copepods, the RGB values of the G_5_ and 50-d PSB populations demonstrated no significant deviation from those of the G_0_ population (Table 1). Regarding the frequency distribution, individuals with RGB values spanning from 160 to 180 occurred frequently and consistently in the populations of all generations (Figs. 3 and 4).

**Table 1:** RGB value (mean ± standard deviation) of the LBS and DBS male and female in every generation. The different letters (a,b,c,d,e) above each bar represent significant differences (p < 0.05) among generations. PSB: post selective breeding.

Generation	LBS		DBS	
	Male	Female	Male	Female
G_0_	163.9 ± 13.1^a^	162.2 ± 14.6^a^	163.9 ± 13.1^a^	162.2 ± 14.6^a^
G_1_	165.8 ± 7.7^a^	163.7 ± 8.4^a^	147.2 ± 8.8^b^	145.2 ± 8.4^b^
G_2_	158.5 ± 7.3^b^	150.9 ± 11.9^b^	131.2 ± 10.1^c^	123.7 ± 10.0^c^
G_3_	161.2 ± 6.1^ab^	159.9 ± 7.3^a^	126.1 ± 11.2^ cd^	120.3 ± 9.2^c^
G_4_	165.8 ± 7.7^a^	162.7 ± 7.0^a^	117.3 ± 9.8^e^	109 ± 11.3^d^
G_5_	164.7 ± 5.0^a^	160.5 ± 6.5^a^	121.5 ± 14.1^de^	108.8 ± 15.0^d^
50-d PSB	163.0 ± 6.2^ab^	164.9 ± 6.7^a^	130.0 ± 16.0^c^	113.5 ± 16.1^ cd^

**Figure 5:**
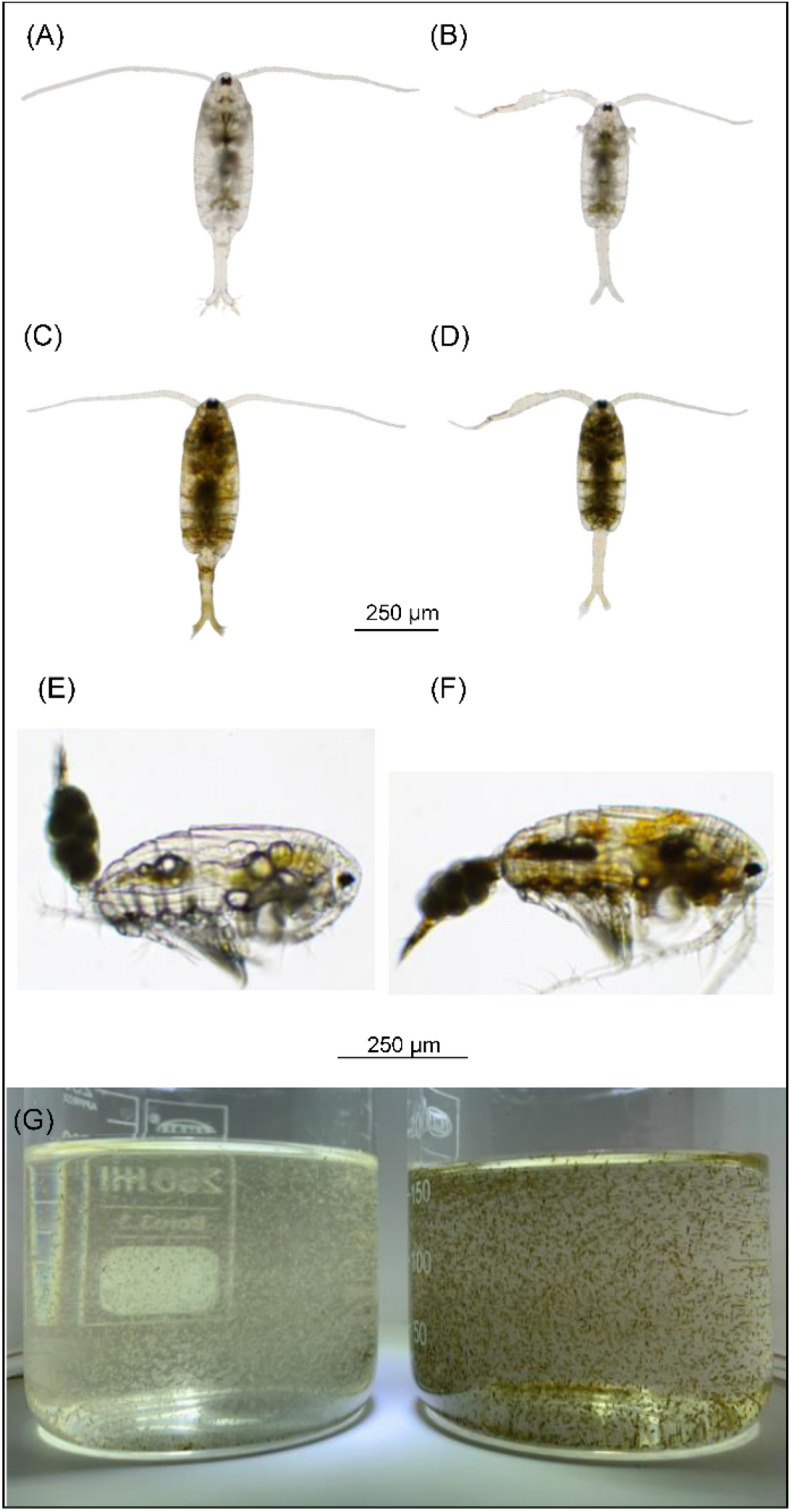
*P. annandalei* individuals and populations of different strains, (**A**) LBS female; (**B**) LBS male; (**C**) DBS female; (**D**) DBS male; (**E**) LBS ovigerous female, lateral side; (**F**) DBS ovigerous female, lateral side; (**G**) LBS population, left and DBS population, right.

Conversely, the DBS copepods showed notable shifts in RGB values. The G_5_ and 50-d PSB populations exhibited significantly lower RGB values in comparison to the G_0_ population (Table 1). Remarkable changes were evident in the frequency distribution of RGB values among the DBS populations across successive generations. The range of the top 10% distributed RGB value reduced from 170 to180 at G_0_ to 110 to140 at G5 and 50-d PSB (Figs. 3 and 4). The genetic gain values elucidate the trend within the DBS population (Table 2). Notably, higher genetic gains were recorded in the first two generations (− 10.49% to − 15.17%), whereas the three last generations exhibited comparatively lower genetic gains (− 0.1% to − 9.3%). Overall, based on the imagery analysis and visualization (Fig. 5), the body color of the DBS copepods appeared darker compared to the LBS copepods after the selective breeding program.

**Table 2:** Genetic gain (%) of RGB value of the LBS and DBS populations across generations.

Genetic gain (%)				
Generation	LBS		DBS	
	Male	Female	Male	Female
G_0_-G_1_	1.15	0.92	− 11.34	− 10.49
G_1_-G_2_	− 4.40	− 7.81	− 10.86	− 15.17
G_2_-G_3_	1.70	5.62	− 3.88	− 2.74
G_3_-G_4_	2.85	1.72	− 6.97	− 9.39
G_4_-G_5_	− 0.66	− 1.37	− 0.18	0.20

#### Reproductive performances

**Figure 6:**
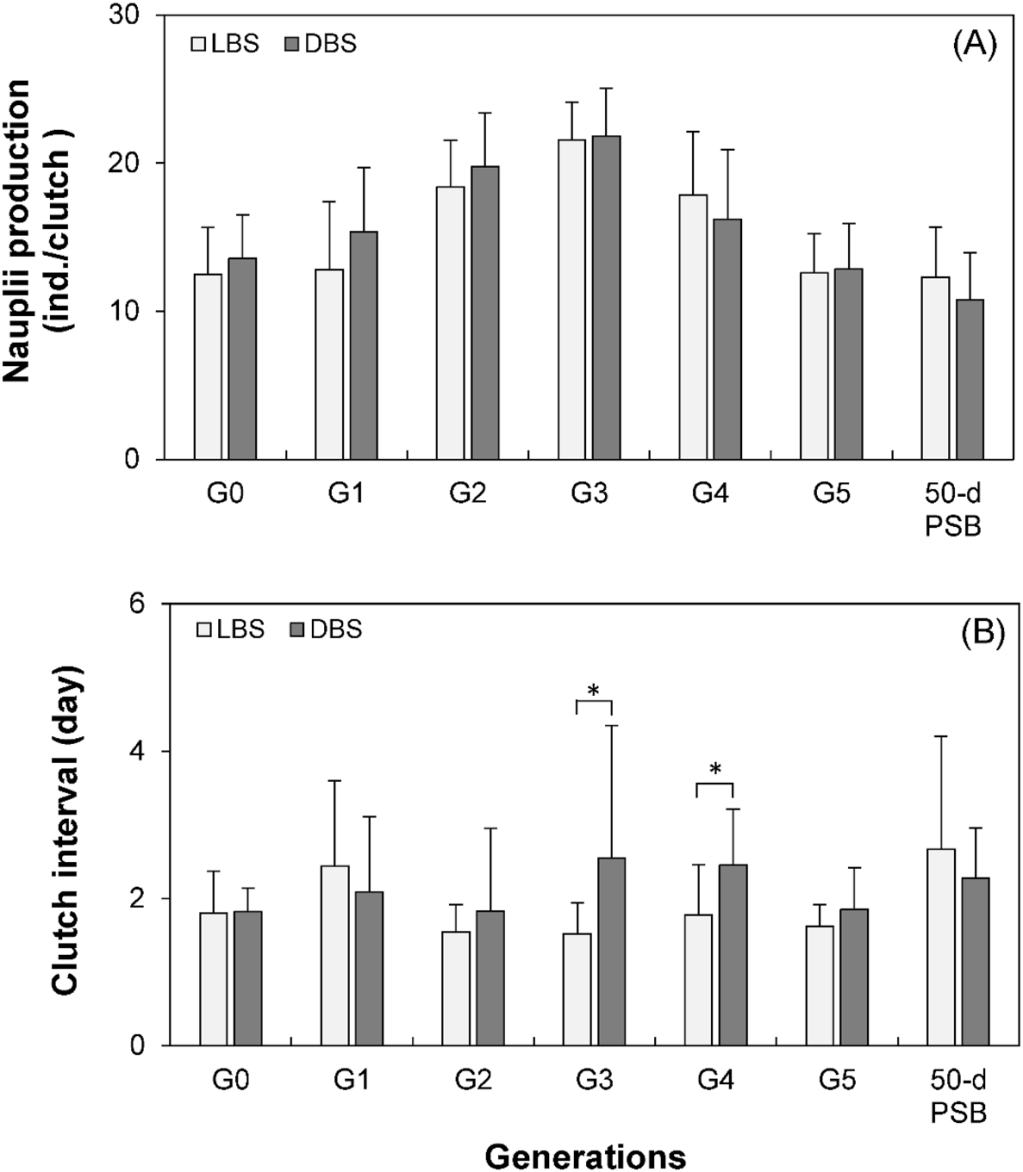
(**A**) Nauplii production, and (**B**) clutch interval of *P. annandalei* DBS and LBS females across generations. PBS: post selective breeding.

While nauplii production fluctuated across successive generations, no significant differences were found between the strains within each generation (Fig. 6). In terms of clutch interval, no noteworthy disparity was detected between the strains across generations, except for the G_3_ and G_4_ generations, where a significant difference surfaced (Fig. 6).

#### Astaxanthin content

**Figure 7:**
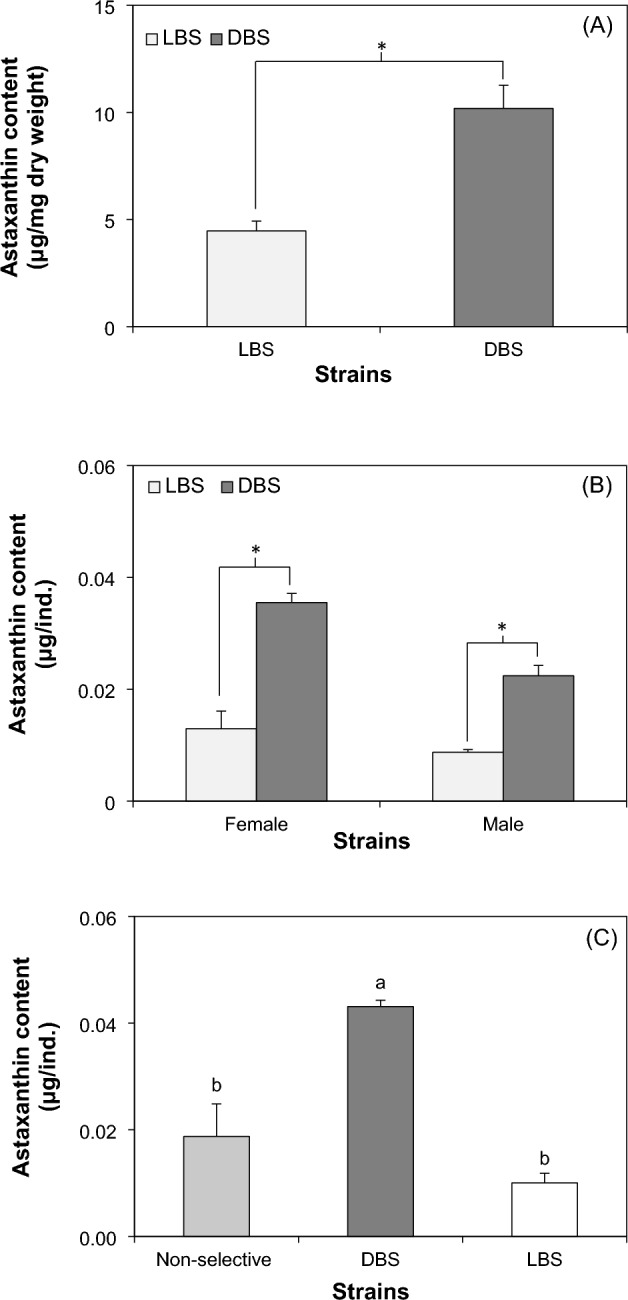
Astaxanthin contents of *P. annandalei*, (**A**) astaxanthin content per dry biomass of the LBS and DBS copepod at 50 days post selective breeding; (**B**) astaxanthin content per male and female individual of the LBS and DBS copepod at 50 days post selective breeding; (**C**) astaxanthin content per individual (non-sexual specific) at 20 months post selective breeding. Asterisk (*) and letters (**a**, **b**) indicate significant differences.

In terms of astaxanthin content, a substantial contrast emerged between the DBS copepods and LBS copepod at 50-d PSB. Specifically, the 50-d PSB DBS copepods exhibited significantly higher astaxanthin concentration, measured at 10.19 μg/mg dry weight, in comparison to the LBS copepods with an astaxanthin concentration of 4.47 μg/mg dry weight (Fig. 7A). Further assessing astaxanthin content on a per individual basis (Fig. 7B), the 50-d PSB DBS copepods (female: 0.036 μg/ind; male: 0.022 μg/ind) showcased significantly higher astaxanthin compared to the LBS copepods (female: 0.013 μg/ind; male: 0.009 μg/ind). In the extended analysis, the DBS copepod (Fig. 7C) also showed significant higher astaxanthin concentration (0.04 μg/ind) than the LBS (0.01 μg/ind) and non-selective strain (0.02 μg/ind) 20-month PSB.

## Discussion

In this study, the laboratory-cultured strain of copepod *P. annandalei*, originally isolated from brackish waters in Tungkang, southern Taiwan, underwent a domestication process over a decade. Intriguingly, a small subset of approximately 2% of individuals in the stock culture exhibited dark body coloration, denoted by an RGB value below 120. This novel observation of body color variation in *P. annandalei,* not documented in either wild or aquacultured populations before, suggests that the extended laboratory culture might have induced these distinct color traits, aligning with findings in other crustaceans, subjected to domestication or artificial selection^49,50^.

The trade-off between predation stress and copepod body colors has been noted in several studies^51^. Typically, highly pigmented copepods are more susceptible to predator detection in shallow waters^52^. If there are no predators in the scenario, as in the case of the mono-species copepod in this study, dark-colored individuals would not be captured and could survive and reproduce within the population. Therefore, a reduction in predators could potentially promote the prevalence of highly pigmented individuals within the long term copepod stock culture. Conversely, the astaxanthin content in copepod is positively correlated with light exposure. In their natural habitat, the semi-benthic *P. annandalei* tend to stay on the bottom of the water column^46,53^, where the light intensity is low. Conversely, in the laboratory conditions, characterized by clear water and transparent containers, the copepods could have experienced higher light intensity. This environmental difference might contribute to the emergence of dark individuals in our stock culture. While phenotypic plasticity is known to play an important role in regulating pigment content in zooplankton under strong seasonality^31,54^, the stable conditions of our *P. annandalei* stock culture suggest that the observed pigmentation enhancement in the dark-colored *P. annandalei* seems to be a result of genetic variation, rather than short-term and environmental-stimulated phenotypic plasticity.

Variations in body coloration among individuals are frequently observed in crustaceans. Weaver et al.^55^ identified body color variations in anchialine shrimp (*Halocaridina rubra*) from different locations, with individuals of higher astaxanthin content producing offspring exhibiting elevated astaxanthin levels, and the body color remained stable through a 14-year laboratory culture. Additionally, body color diversity in the ornamental shrimp *Neocaridina denticulata* has been linked to specific genes, hinting at potential biomarkers for the genetic selection of specific body colors or patterns^50^. These findings resonate with our observations during the selective breeding of *P. annandalei*, suggesting that traits such as body color and astaxanthin content can be genetically inherited in crustaceans. Future studies, such as cross breeding programs, could be conducted to investigate the genetics mechanisms involved in copepod astaxanthin metabolism.

Previous research has demonstrated that the efficiency of astaxanthin bioconversion in copepods is influenced by dietary astaxanthin precursors. For instance, dietary supplementation with zeaxanthin and canthaxanthin has been shown to elevate astaxanthin content in copepods^14^. Conversely, a decline of astaxanthin in copepod could occur when they are fed with carotenoid-deficient diets such as yeast^14,56^. In this study, microalga *I. galbana,* rich in carotenoids including fucoxanthin and β-carotene^57^, was used as a dietary source, providing astaxanthin precursors for both DBS and LBS populations. The strain-specific variations in astaxanthin content might stem from differential capacity in astaxanthin bioconversion and accumulation. The biosynthesis pathway of astaxanthin could involve many steps of enzymatic activities that produce a diverse groups of precursor metabolites (β-carotene, echinenone, and canthaxanthin), yet it is scarcely studied in copepod species^51,58^. In the future, the comparison of biosynthesize enzymes or relative gene expression levels between the two *P. annandalei* strains should be investigated using molecular biotechnology techniques, such as transcriptomic or metabolomic analysis, to uncover the molecular mechanisms that cause the variation of their astaxanthin concentration.

Studies have linked enhanced pigmentation to increased egg production rates in copepod^52^, and positive correlations between astaxanthin content and productivity in aquatic animals have been reported^34,59^. Interestingly, our findings differ, as DBS females displayed relatively longer clutch intervals across most generations. This discrepancy could be attributed to energy allocation shifts in copepods, possibly stemming from the allocation of more energy towards astaxanthin synthesis and accumulation, resulting in reduced reproductive frequency. However, there are many other life traits affecting the overall productivity of copepod production, including offspring survival rate, life span, developmental time, stress tolerance and cannibalism^60,61^. The various life traits of LBS and DBS copepods should be studied to further validate their productivity in the mass culture condition.

In this study, the high-astaxanthin DBS *P. annandalei* strain was established through selective breeding. This selective strain displays significantly rich astaxanthin content compared to levels reported in other copepod species (as shown in Table 3), indicating their great potential as live feed for marine or brackish larviculture. Furthermore, these selected strains could serve as experimental models for delving into various aspects of copepod physiological and ecological studies, including mechanisms of astaxanthin bioconversion, antioxidative capacities, and the role of body-color-based prey-predator interactions.

**Table 3:** Comparison of astaxanthin content in different copepod species.

Species	Range of astaxanthin content (μg/mg dry weight)	References
*Pseudocalanus acuspes*	0.22–0.32	Łotocka et al. (2004)
*Nitokra lacustris*	0.19–0.65	Rhodes (2007)
Mixture of *Acartia grani, Centropages hamatus,* and *Eurytemora affinis*	0.41–1.42	van der Meeren et al. (2008)
*Tigriopus californicus*	4.23	Weaver et al. (2018)
*Calanus finmarchicus*	0.14–0.27	Th Eysteinsson et al. (2022)
LBS *P. annandalei*	4.47	Present study
DBS *P. annandalei*	10.19	Present study

## Data Availability

The data that support the findings of this study are available on request from the corresponding author J.Y.P.
